# The habitat use of ariid catfishes in coral reefs in northeastern Brazil

**DOI:** 10.1111/jfb.70654

**Published:** 2026-09-18

**Authors:** Romullo Guimarães Lima, Bruno Eleres Soares, Clecio da Silva Pereira, Wesley Ruan Fernandes Bezerra, Paloma Rocha da Silva, Livian Kertleen Pôrto Jesuino, Mariana Vitória dos Santos Gemaque, Carlos Alberto Cavalcanti Soares, Ana Lúcia Vendel, Alexandre P. Marceniuk

**Affiliations:** ^1^ Departamento de Biologia Universidade Federal do Ceará Fortaleza Brazil; ^2^ Institute of Environmental Change & Society University of Regina Regina Saskatchewan Canada; ^3^ Programa de Pós‐Graduação em Ecologia Universidade Federal do Rio de Janeiro, UFRJ Rio de Janeiro Brazil; ^4^ Paraíba State University‐UEPB Centre for Applied Biological and Social Sciences CCBSA, Campus V João Pessoa Brazil; ^5^ Paraíba State University‐UEPB Centre for Applied Biological and Social Sciences CCBS, Campus I Campina Grande Brazil; ^6^ Federal Institute of Paraíba/PREAMAR João Pessoa Brazil; ^7^ Postgraduate Program in Ecology and Conservation Paraíba State University Campina Grande Brazil

**Keywords:** biogenic reef, feeding, length‐weight relationship, population dynamics, reproduction

## Abstract

Ariidae species occur across a wide range of habitats from freshwater to fully marine environments and are typically associated with soft‐bottom substrates. However, they are sometimes associated with hard‐bottom substrates such as coral reefs, but such association is poorly documented. This study describes the occurrence and habitat use of six ariid species in the coral reef of Penha Beach, northeastern Brazil. We deployed gill nets monthly alongside the coral reef over a year between 2023 and 2024, and we sampled 256 individuals representing six ariid species. *Bagre filamentosus*, a marine catfish, was the most abundant species throughout the year. Species composition varied seasonally, and an RDA analysis showed that variation in species abundances was associated with precipitation and air temperature, with *Cathorops spixii* occurring primarily during the wet season. *B. filamentosus* and *Sciades proops* showed male‐biased sex ratios across all seasons (exact binomial tests), and males of *B. filamentosus* exhibited higher mass gain per unit length during the dry season (t‐test). Diet composition varied among months and according to the species' habitat affinity, although effect sizes were small and seasonal differences were weak. These findings suggest that ariid catfishes use coral reef systems as opportunistic foraging and refuge habitats, with habitat use probably linked to their life cycle and the connections between coral reefs and estuaries.

## 1 INTRODUCTION

The family Ariidae (Siluriformes) comprises more than 150 species of sea catfishes found in tropical and subtropical coastal environments worldwide, across a remarkable range of salinity regimes, from freshwater to fully marine habitats (Betancur‐R, 2009; Marceniuk et al., 2024). Sea catfishes are among the few otophysan fishes that have secondarily colonized marine environments, with at least 10–15 independent marine‐to‐freshwater transitions worldwide, making them a compelling model for studying habitat transitions in fishes (Betancur‐R, 2010; Rincon‐Sandoval et al., 2024). These species have a life history deeply tied to soft‐bottom, high‐turbidity environments. Euryhaline and eurythermal, they occupy salinity gradients from oligohaline estuarine channels to open coastal waters, with spatial distribution within estuarine systems governed by temperature and salinity gradients (Dantas et al., 2012; Pusey et al., 2020). Their habitat affinities vary among ariid species, with some species primarily occurring in marine environments (e.g., *Bagre bagre* and *Bagre filamentosus*) and others being more strongly associated with estuarine environments (e.g., *Cathorops spixii*) (A. P. Marceniuk et al., 2024). As generalist benthic predators, they feed primarily on crustaceans, fish, molluscs and plant material, with high dietary plasticity across habitats (Mendes & Barthem, 2010; Simier et al., 2021; Yànez‐Arancibia & Lara‐Dominguez, 1988).

Understanding how ariids use coastal habitats requires considering not only how environmental gradients shape their distribution, but also their unique reproductive strategies. Their reproductive biology is the most distinctive characteristic of the family. All ariid species practice obligatory paternal oral incubation: following spawning, during the wet season (Mourão & Nordi, 2003; Pinheiro et al., 2006), males carry fertilized eggs in the buccal cavity throughout embryonic and early post‐embryonic development, ceasing to feed during the entire brooding period (Bruton, 1996; Simier et al., 2021). This strategy results in very low absolute fecundity (fewer than 50 eggs per female in most species) and limited dispersal capacity, a consequence of the absence of a pelagic larval stage (Correia et al., 2020; Gomes et al., 1999). Although typically associated with soft‐bottom, estuarine and mangrove environments, ariid catfishes have been recorded in artificial reef systems along the southeastern Brazilian coast, where they rank among the most representative families in terms of abundance and occurrence (Gatts et al., 2014; Lima et al., 2020). On the other hand, previous reef surveys in northeastern Brazil did not record any Ariidae species (Honório et al., 2010), although those surveys were based primarily on underwater visual censuses, which may fail to detect cryptic, nocturnal or reef‐associated transient catfishes. In fact, when recorded in hard‐substrate structures, ariids are usually transient species that exploit the hard‐substrate habitats as foraging grounds for benthic prey and temporary refuge (Lima et al., 2020).

Northeastern Brazil provides an interesting setting to document associations between ariids and coastal habitats, particularly reef habitats, because seasonal freshwater discharge and the close spatial proximity of estuarine and reef environments create a dynamic coastal habitat mosaic that shapes the distribution of marine‐ and estuarine‐associated species. Ariid catfishes are common components of coastal and estuarine fish assemblages in Northeastern Brazil, which harbour multiple species varying in their habitat affinities (A. Marceniuk et al., 2021; Medeiros et al., 2018; Vendel et al., 2022).

In this study, we describe the occurrence, habitat use, feeding ecology and reproductive condition of six ariid catfish species in the biogenic reefs of Penha Beach, Paraíba state, over a full annual cycle. Specifically, we ask (i) how species use this reef system during the year; (ii) how environmental variables are associated with species presence in the reef system; (iii) whether sex ratios differ from 1:1 expectations across seasons; (iv) whether feeding strategies reflect foraging on hard‐substrate prey versus soft‐bottom prey and (v) whether species with different ecological affinity (estuarine vs. marine) exploit dietary reef resources differently.

## 2 METHODS

### 2.1 Study area

The coral and biogenic sandstone reefs of the Paraíba coast, including those at Penha Beach, in João Pessoa municipality, are shallow fringing reef systems resting on a Quaternary sandstone foundation consolidated by calcareous material produced primarily by the green algae *Halimeda* and encrusting coralline algae (Maida & Ferreira, 1997). They are adjacent to estuarine systems and exposed to chronic freshwater and nutrient inputs from continental drainage and submarine groundwater discharge (SGD) (Maida & Ferreira, 1997; Massei et al., 2023; Santos et al., 2021; Silva et al., 2022) (Figure 1). In a region dominated by the Brazil Current that flows southward and transports warm Tropical Water (26.0–29.5°C; salinity 36.5–37.1 psu) along the shelf edge, local mean currents weaken substantially during the wet season (~0.06 m/s) and tidal forcing becomes responsible for up to 75% of cross‐shore current variance, sharply increasing water residence time at the reef precisely when continental freshwater and nutrient inputs reach their annual maximum (Santos et al., 2021). This area receives input of untreated domestic and industrial effluents from Gramame River and Cabelo River (Figure S 6) (Massei et al., 2023; Silva et al., 2022) which might increase eutrophication and algal biomass in the region (Fabricius, 2005; Hughes et al., 2007).

**FIGURE 1:**
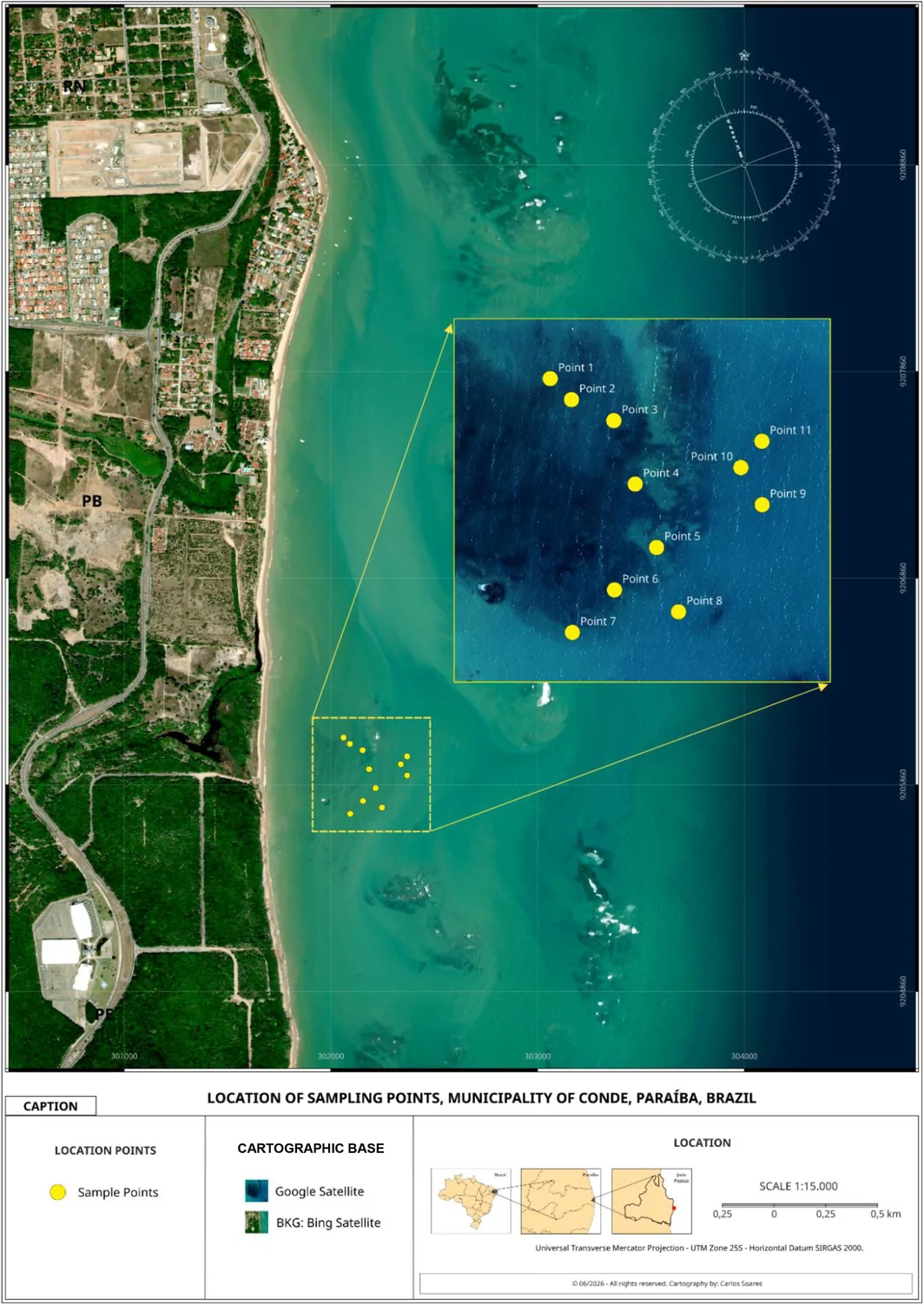
Map of the sampling points in Penha beach, Paraíba, northeast of Brazil.

### 2.2 Sampling design

Fish were collected under authorization from the environmental Brazilian agency through licences granted to the Ichthyology Laboratory at the Paraíba State University. Sampling expeditions were carried out monthly between September 2023 and August 2024 at Penha beach using gill nets (3 cm mesh size, 320 m length). Nets were deployed around the coral reef for 12 h (6 pm to 6 am), except in October when sampling lasted 24 h. Sampling locations were distributed around the same reef structure and were separated by less than 250 m. Expeditions were carried out in both the dry (September–February) and the wet (March–August) seasons. Environmental data (precipitation, maximum wind gust velocity, air temperature) were obtained from the Instituto Nacional de Meteorologia (INMET). We selected precipitation and air temperature as proxies of environmental variation because freshwater discharge increases during rainy periods and is known to influence salinity in coastal systems (Araújo, 1988; Barletta et al., 2005; Da Silva et al., 2022). Maximum wind gust velocity was included because wind forcing can influence stratification and tidal dynamics, and the extent of freshwater plumes (Dos Santos et al., 2010; Paiva et al., 2025). For each sampling event, environmental variables were calculated as the mean values from 10 days before to 10 days after the sampling date.

### 2.3 Ethics statement

All procedures complied with IBAMA/ICMBio regulations under permits n. 18,623–1, n. 31,000–1 and 90,711. Fish were captured using gillnets and were dead at the time of retrieval. Consequently, no euthanasia procedures were performed, and all specimens were processed post‐mortem in the laboratory.

### 2.4 Laboratory analysis

Fishes were identified following Marceniuk et al. (2021, 2023). Standard length and total weight were measured using an ichthyological rule or calliper (0.1 cm precision) and a scale (0.1 g precision), respectively. To characterize the reproductive features, sex was described visually by observing individuals' gonads and gonad weight was measured using a precision scale (0.02 g precision). All individuals were then dissected, and their stomachs were removed for gut content analysis. Stomach contents were identified to the lowest taxonomic level possible following Fricke et al. (2026) and quantified using the volumetric procedure (Albrecht & Pellegrini‐Caramaschi, 2003; Hyslop, 1980). Only stomachs containing items were used for diet analyses.

### 2.5 Data analysis

We ran a redundancy analysis to understand the relationship between monthly species abundance and the environmental variables precipitation, maximum wind gust velocity and air temperature. Species abundance was standardized using the Hellinger method, and environmental variables were standardized to mean 0 and standard deviation 1. We applied an analysis of variance (ANOVA) to test if the variables affected occurrence.

We tested whether sex ratios differed from the expected 1:1 proportion for the three most abundant species using exact binomial tests separately for each combination of species and season. Differences in sex ratio between seasons were assessed using Pearson's Chi‐squared test for *B. filamentosus* and Fisher's Exact test for *N. luniscutis* and *S. proops* due to small sample sizes (<5 individuals) in some periods. For *B. filamentosus*, we fitted linear regressions relating log‐transformed standard length and total weight separately for males and females. We compared the length‐weight relationship parameters between the wet season and the dry season using an analysis of variance (ANOVA).

To describe each species' diet, we calculated the index of alimentary importance for stomachs with content (IA_*i*_, Kawakami & Vazzoler, 1980) following the formula:

$${\mathrm{IA}}_{i}=\frac{{\mathrm{FO}}_{i}*V_{i}}{\sum_{i=1}^{n}{\mathrm{FO}}_{i}*V_{i}}$$

where FO_*i*_ is the occurrence frequency of the item *i* across stomachs and *Vi* is the percentual volume of the item compared to the total volume.

To evaluate whether diet composition varied according to species habitat affinity (estuarine: *Cathorops spixii;* marine: *Bagre bagre* and *Bagre filamentosus*; or intermediate: *Notarius luniscutis*, *Notarius parmocassis* and *Sciades proops*), month and season (wet vs. dry), we performed a Permutational Multivariate Analysis of Variance (PERMANOVA) using Bray–Curtis dissimilarities based on differences in the percentual volume of each food item. These analyses were followed by PERMDISP analyses to test if differences were due to the position of the centroid or the dispersion of each group.

All analyses were performed in R environment (R Core Team, 2026), using functions from *vegan* (Oksanen et al., 2026) and *mgcv* (Wood, 2011) to run analyses, with support of functions from *tidyverse* (Wickham et al., 2019), *readxl* (Wickham & Bryan, 2026) and *PerformanceAnalytics* (Peterson & Carl, 2026) to manipulate the data and from *ggplot2* (Wickham, 2016), *ggrepel* (Slowikowski, 2026), *ggtext* (Wilke & Wiernik, 2022) and *patchwork* (Pedersen, 2025) to plot graphics.

## 3 RESULTS

We sampled a total of 256 individuals belonging to six species of the family Ariidae. They were all adults ranging from 19 to 46 cm (mean = 22.7 cm, sd = 4.9 cm). *Bagre filamentosus* Pellegrin, 1924 was the most abundant species on the reef, with 144 individuals recorded throughout the year, followed by *Sciades proops* (Valenciennes, 1840) with 35 individuals and *Notarius luniscutis* (Valenciennes, 1840) with 30 individuals (Figure S1).

The temporal variation in abundance throughout the year differed among species (Figure S2). Variation in species composition changes was associated with air temperature (*F* = 3.474, *p* = 0.019, RDA1 correlation: −0.70, RDA2: 0.02) and precipitation (*F* = 2.84, *p* = 0.048, RDA1: −0.15, RDA2: −0,80) (Figure 2). *Bagre bagre* (Linnaeus, 1766) peaked in October and declined after, being sampled mainly when air temperature was lower and winds stronger; B. *filamentosus* remained the dominant species throughout the year, with consistently high abundance across both precipitation periods; whereas *Cathorops spixii* (Agassiz, 1829) was recorded mainly during high precipitation periods; *N. luniscutis* showed higher abundance during wet months, being absent when temperatures were higher with low precipitation; *Notarius parmocassis* (Valenciennes, 1840) was sampled in two warm months and *S. proops* occurred at relative low abundances year‐round (Figures 2, S2, Table S1).

**FIGURE 2:**
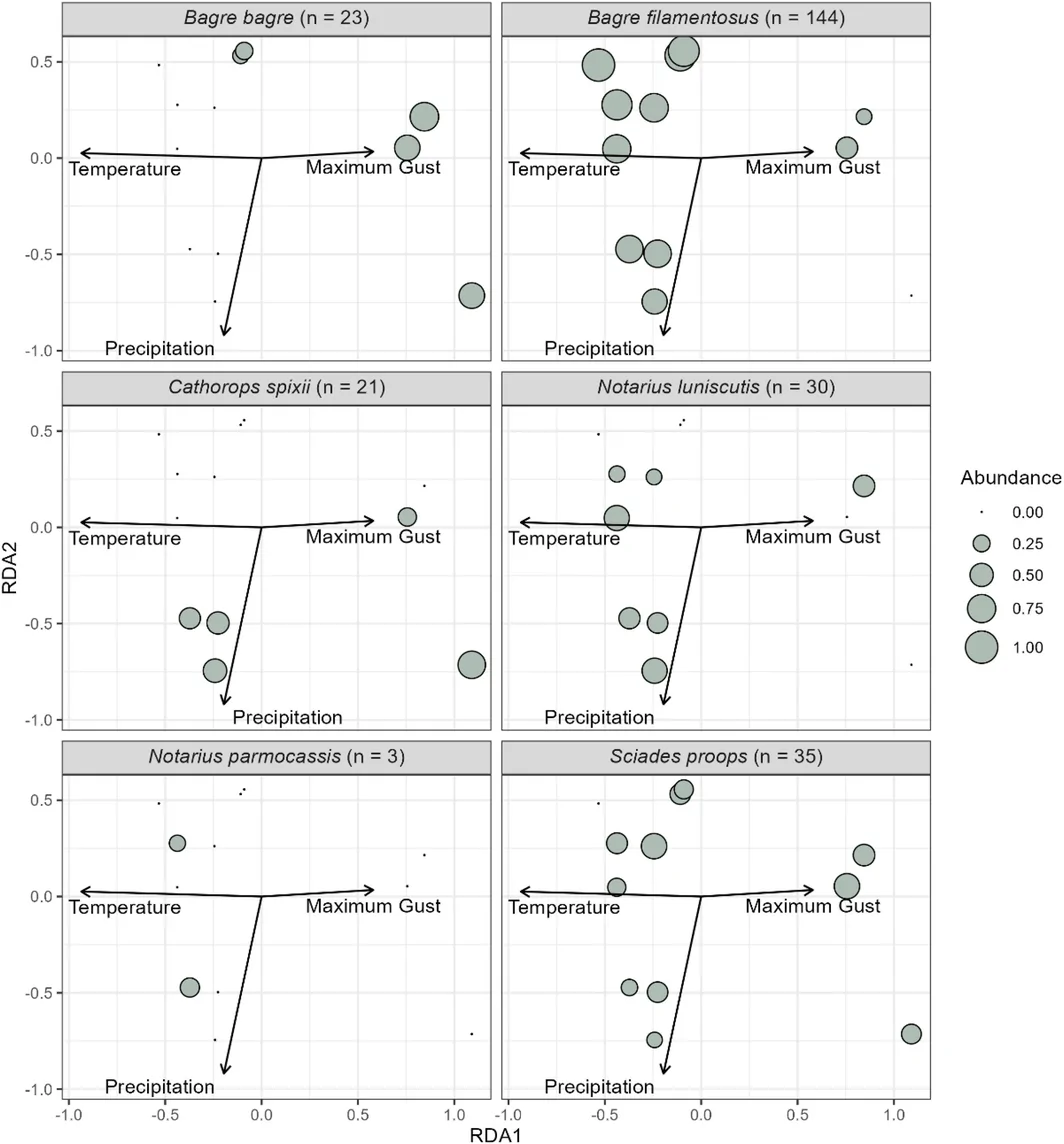
Redundancy Analysis (RDA) based on species abundance and environmental values across sample units. For each grid of the panel, the position of the point is the score of the sample unit in the RDA and the size of the point is the Hellinger standardized abundance of the species. The first two axes explain 46.5% of the variation (RDA1: 27.2%, RDA2: 19.4%).

Sex ratios did not differ between wet and dry seasons for any of the most abundant species (*B. filamentosus*: *p* = 0.1382, *N. luniscutis* and *S. proops: p* = 1). For the less abundant species, the test was not possible due to the small number of individuals. In both seasons, males were more frequent than females in *B. filamentosus* (male: female ratios: 2.23:1 dry and 4.5:1 wet season) and *S. proops* (4.3:1 dry and 3.25:1 wet). In contrast, *N. luniscutis* showed no significant deviation from a 1:1 sex ratio (dry season *p* = 1, wet season *p* = 0.15) despite a higher number of females (Figure 3).

**FIGURE 3:**
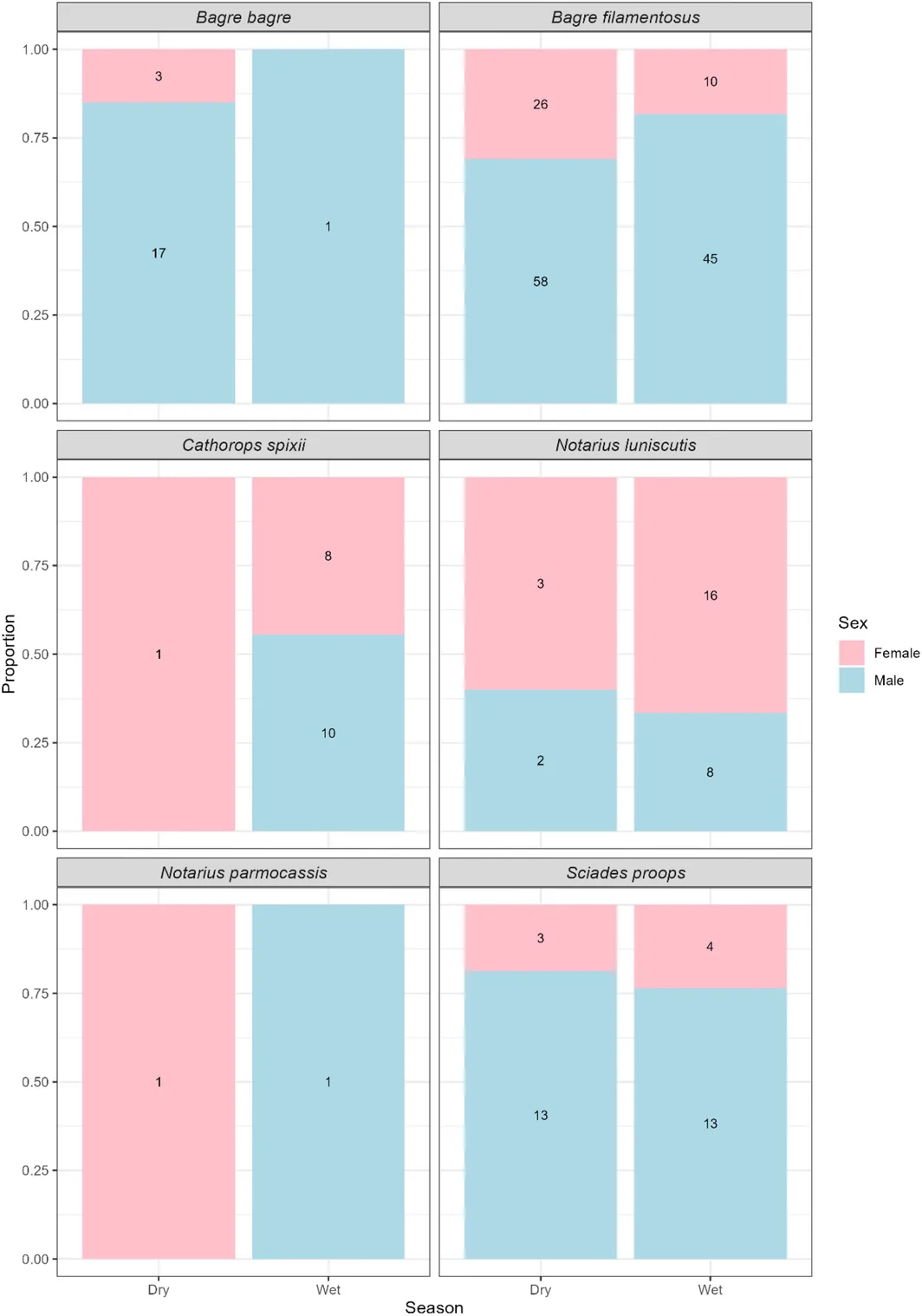
Proportion of males and females for the six sampled species in the different seasons. The number inside bars represents the number of individuals. Only species with more than one individual in both dry and wet seasons were tested for differences in 1:1 ratio and between seasons.

The length‐weight relationship of *B. filamentosus* males differed between seasons (*p* = 0.0165). Males exhibited positive allometric growth in the dry season (*b* = 3.396, *a* = 1.806 × 10^−6^) and negative allometric growth in the wet season (*b* = 2.7735, *a* = 5.383 × 10^−5^) (Figure S3). No seasonal differences in the allometric coefficient were detected for females (*b*_wet_ = 3.4, *b*_dry_ = 3.35, *p* = 0.862; *a*_wet_ = 1.88 * 10^−6^, *a*_dry_ = 2.48 * 10^−6^).

Diet composition varied among months (PERMANOVA; *p* = 0.034), but the effect size was small (*R* ^2^ = 0.08). This variation was driven by differences in composition rather than group dispersion (PERMDISP; *F* = 0.8, *p* = 0.641), and differences were not strongly associated with wet and dry periods (*p* = 0.303). Similarly, habitat affinity explained a significant but limited portion of the variation in diet composition (*p* = 0.001, *R* ^2^ = 0.09), but this difference was not driven by group dispersion (*F* = 0.58, *p* = 0.571). Pairwise permutation analysis shows that diet changed between marine vs. estuarine species (*p* = 0.021, *R* ^2^ = 0.02), estuarine vs. intermediate (*p* = 0.01, *R* ^2^ = 0.14) and marine vs. intermediate (*p* = 0.01, *R* ^2^ = 0.08).

*B. bagre* (*n* = 18 stomachs), with primarily association with marine environments, exploited both hard‐ and soft‐substrate resources. In the dry season, individuals consumed a broader range of food items, including plants, crustacean (mostly Brachyura) and fish, with a greater contribution of hard‐substrate food items. In the wet season, its diet was dominated by crustaceans associated with soft substrates (mostly Brachyura) and fish (e.g. Clupeidae). Polychaeta were present in the diet in both periods. *B. filamentosus* (*n* = 117, primarily marine association) fed across both substrate types, with most of their diet composed by hard‐substrate food item, vegetal matter (IAI ≅ 80%), in both seasons. *C. spixii* (*n* = 11, primarily estuarine association) fed almost exclusively on hard‐substrate resources, being vegetal matter the dominant food item (IAI ≅ 95%). *N. luniscutis* (*n* = 18, intermediate association) fed primarily soft‐substrate food items, with its diet being mainly composed of crustaceans, with Brachyura dominating during the wet season. In the dry season, both Peneidae shrimps and echinoids were important, the latter representing almost 50% of the diet. *N. parmocassis* (*n* = 3, intermediate association) fed mostly on soft‐substrate food items, with crustaceans being the main item in their diet (IAI ≅ 90%). In addition, a seasonal shift in their diet was observed, with Brachyura dominating in the dry period and Peneidae during the wet period. *S. proops* (*n* = 23, intermediate association) fed primarily on soft‐substrate resources, with crustaceans (mostly Brachyura) and fishes being dominant items in both periods (Figures 4, 5, S4 and S5).

**FIGURE 4:**
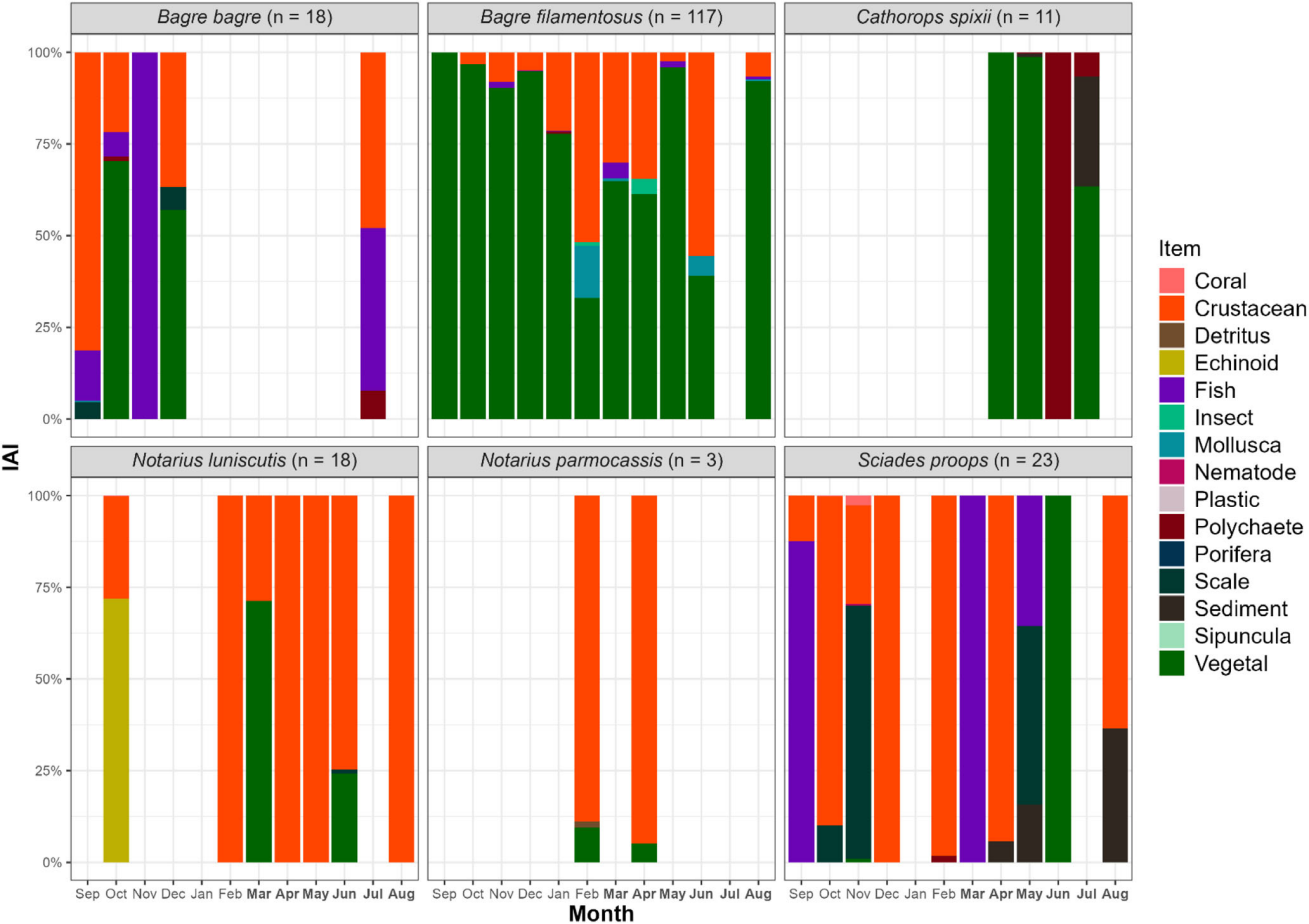
Alimentary index for the species for the different months. Food items were grouped into higher levels. Months in bold represent the wet season.

**FIGURE 5:**
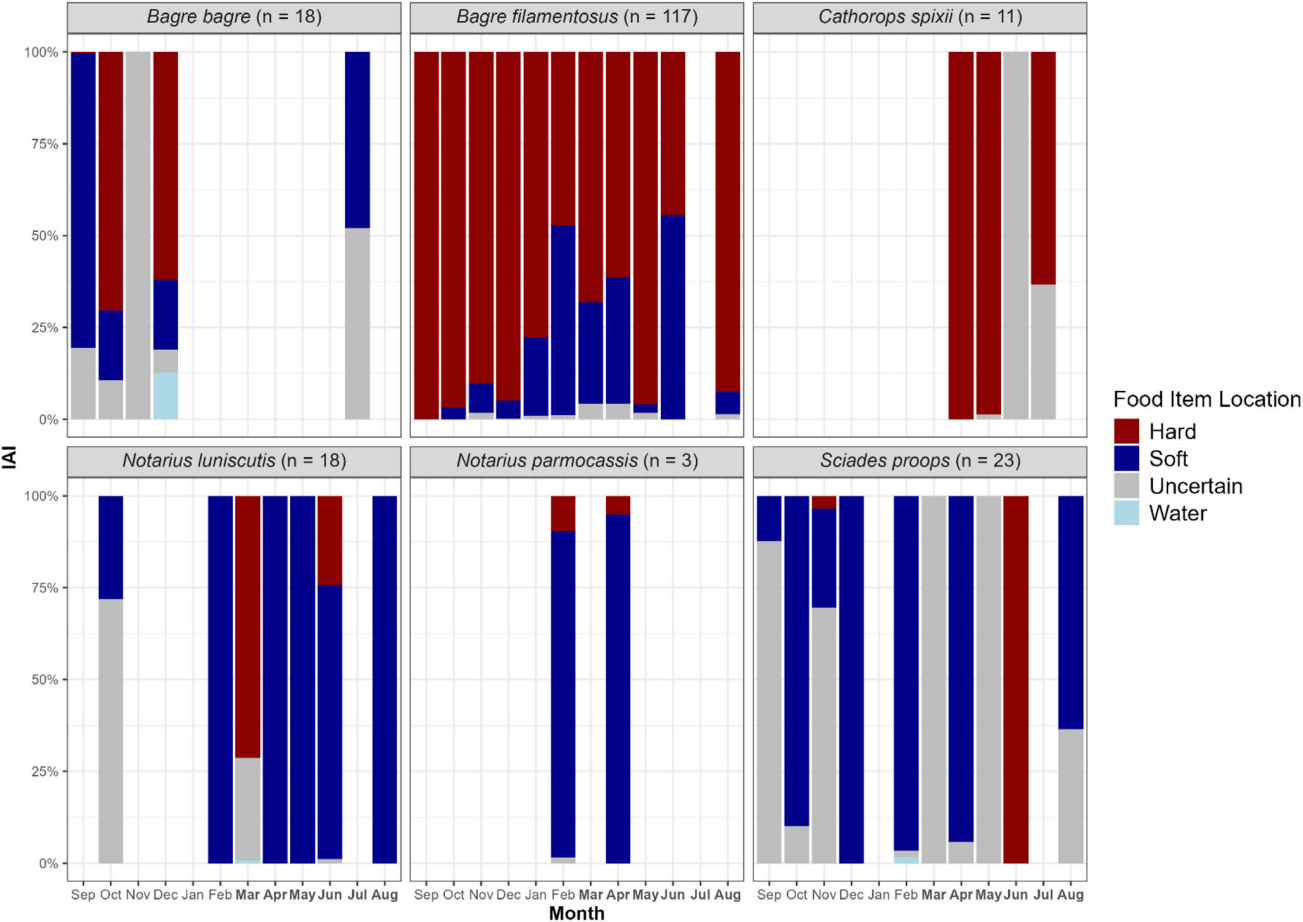
Alimentary index for the species for the different months. Food items were grouped into their location. Hard bottom, soft bottom, water (pelagic) and uncertain. Months in bold represent the wet season.

## 4 DISCUSSION

This study provides the first systematic documentation of the occurrence, feeding ecology and reproductive condition of ariid catfishes at the biogenic reef–soft bottom interface in Northeastern Brazil over an annual cycle. We found that species composition varied seasonally, with changes primarily associated with variation in precipitation and atmospheric temperature. The two most abundant species, *Bagre filamentosus* and *Sciades proops*, showed a consistent male‐biased sex ratio, and males of *B. filamentosus* exhibited higher weight at a given length during the dry season. Diet composition varied among months and among species with different habitat affinities, although effect sizes were small.

Ariid catfishes are commonly associated with soft‐bottom marine or estuarine habitats (Araújo, 1988; Barletta et al., 2005; Gomes et al., 1999; Yànez‐Arancibia & Lara‐Dominguez, 1988), but our results demonstrate a consistent use of hard‐bottom reef environments throughout the year. This pattern might suggest that these habitats constitute a broader coastal habitat mosaic with some importance for catfishes, and the presence of Ariidae species might be related to salinity and distance from estuary (Dos Santos et al., 2010; Gatts et al., 2014). Therefore, changes in distribution between these habitats might not only be related to species life cycle but can also be a consequence of seasonality, when wetter periods extend estuarine conditions to coral reefs, facilitating the occurrence of species usually related to soft‐bottom areas (Da Silva et al., 2022).

The ariid assemblage using the Penha reef is ecologically heterogeneous, comprising species with predominantly marine affinity (*Bagre bagre* and *Bagre filamentosus*), species characteristic of estuarine and coastal soft‐bottom environments (*Cathorops spixii*) and species with intermediate to broad coastal distributions (*Notarius luniscutis*, *Notarius parmocassis* and *Sciades proops*) (Marceniuk et al., 2024). Seasonal variation in species composition was associated with changes in atmospheric temperature and precipitation, which likely reflect variation in freshwater discharge and, consequently, salinity at the reef ecosystem. Although salinity was not measured directly in this study, previous work in the region has shown that seasonal rainfall and continental runoff strongly influence salinity dynamics in adjacent reef habitats (Silva et al., 2022). Therefore, observed patterns are consistent with salinity‐mediated habitat use, particularly for those species with known affinities for estuarine and marine environments. Salinity is a primary driver of species distribution in estuarine and adjacent coastal systems, structuring fish communities along the estuarine‐marine gradient depending on species' environmental tolerances (Barletta et al., 2005; Dantas et al., 2010; Soares et al., 2021). For example, *B. filamentosus* abundance peaked in warmer and drier conditions (high salinity), while estuarine‐associated species such as *Notarius luniscutis* and *Cathorops spixii* were associated with wetter months (low salinity). This pattern is consistent with the ecological preferences described for these taxa in estuarine systems: euryhaline species that are abundant in estuaries increase in abundance in adjacent coastal areas during high‐discharge periods when the reef environment becomes more similar to their preferred habitat (Dantas et al., 2010), while marine species benefit during drier months, when estuarine plumes decrease, increasing reef salinity (Dantas et al., 2010; Lima et al., 2020; Silva et al., 2022).

In addition to environmental variation, the reproductive biology of the ariids might contribute to patterns of reef habitat use. The dominance of males at the reef system throughout the year might be associated with the reproductive strategy of the group. In ariids, males perform oral incubation and experience reduced feeding during brooding periods (Bruton, 1996; Correia et al., 2020). This pattern suggests that the Penha reef might serve as a resting and refuge area after the brooding period, when they are unable to feed and are physiologically compromised. The structural complexity of biogenic reefs may provide shelter from current and predators, offering positive conditions during periods of reduced mobility of feeding (Higgins et al., 2022; Lima et al., 2020). It is also possible that males use this habitat during brooding, but we did not observe this behaviour. Because ariids may release eggs when disturbed during capture, the sampling method used here may underestimate the occurrence of brooding individuals.

Alternatively, because males were present throughout the year, reef use is unlikely to be restricted to the brooding period. An alternative explanation is that the male‐biased sex ratios reflect a spatial segregation between males and females, a pattern consistent with the tendency of ariid females to concentrate in estuarine nursery areas (Dantas et al., 2010; Yànez‐Arancibia & Lara‐Dominguez, 1988). Under this scenario, females may be more associated with estuarine or soft‐bottom habitats, whereas males of *B. filamentosus* and *S. proops* could be more associated with reef habitats; a pattern that cannot be directly tested with the present data. We suggest new samplings in the area, both on marine and estuarine habitats, to clearly understand how the reproductive cycle affects species distribution and these samples should be accompanied by less invasive methods, such as subaquatic images, to evaluate the behaviour and distribution of males during oral incubation.

The males of *B. filamentosus* in the reefs presented higher weight per unit length during the dry season compared to the wet season, suggesting improved body condition in the dry season. This pattern is consistent with a post‐brooding recovery phase, which is reported to happen at the dry season in the region (Pinheiro et al., 2006), during which individuals resume feeding after the energetic costs of oral incubation (Gomes et al., 1999). The structural complexity and benthic prey density of the hard substrate at the reef–soft‐bottom interface may serve as a particularly valuable foraging ground during post‐brooding condition recovery (Gatts et al., 2014; Lima et al., 2020; Watson et al., 2025). The absence of a similar seasonal effect on female body conditions might indicate that the energetic constraints associated with reproduction and oral incubation affect males for a longer period.

Changes in species composition were accompanied by temporal shifts in diet composition. Ariid species used the reef–sandy substrate interface as a foraging ground for mostly benthic prey, particularly crustaceans (Brachyura and Peneidae), plant material and echinoids. The high contribution of plant material in the diet of *B. filamentosus* and *C. spixii* suggests substantial use of reef‐associated primary production, likely from the abundant epilithic and endolithic macroalgae that colonize biogenic sandstone reefs in the region (Silva et al., 2022). This pattern reflects trophic flexibility rather than a diet specialized in plant materials, since *B. filamentosus* and *C. spixii* are known to have an omnivorous diet, feeding on polychaetas, copepods and bivalves (e.g., Denadai et al., 2013).

Diet composition differed significantly among habitat‐affinity groups, with marine‐associated species feeding mostly on plant materials and some hard‐substrate resources, whereas species with intermediate or estuarine affinities fed mostly on soft‐substrate prey. However, habitat affinity explained only a limited proportion of dietary variation (low *R* ^2^), thus observed differences may largely reflect species‐specific feeding habitats rather than broad ecological categories. Temporal variation in diet composition was small and likely reflected seasonal fluctuations in prey availability rather than changes in feeding strategy. Seasonal shifts in the relative importance of prey such as Brachyura and Peneidae are known in tropical coastal systems (Yànez‐Arancibia & Lara‐Dominguez, 1988) and are reported to affect such food webs (Lobato et al., 2018).

Taken together, our results suggest that the Penha reef might be a secondary habitat within the broader estuarine‐coastal system for ariid catfishes. Its use by these catfishes seems to be associated with three factors. First, the physical proximity of the reef to adjacent estuarine systems (Gramame River), combined with the turbid and reduced‐salinity conditions typical of biogenic sandstone reefs in Northeastern Brazil, creates an environment that is ecologically accessible to euryhaline, turbidity‐tolerant ariid species (Dantas et al., 2010; Silva et al., 2022). Second, the hard‐substrate complexity of the reef provides structural refuge and high benthic prey concentration to brooding males and post‐brooding, recovering individuals (Higgins et al., 2022; Lima et al., 2020). Third, the opportunistic generalist foraging strategy of the family enables them to exploit reef‐associated prey, such as crustaceans, echinoids and macroalgae, regardless of their primary ecological affinity (Mendes & Barthem, 2010; Simier et al., 2021). Therefore, these catfishes seem to use the biogenic reef facultatively, integrated into the broad coastal ecosystem. Although our data allow us to make interesting inferences regarding fish distribution patterns, we understand that more samples comprising higher spatial and temporal scales are needed to confirm these findings.

From a conservation perspective, the consistent association of ariid catfishes with the Penha reef highlights the importance of habitat connectivity in coastal systems. The Penha reef provides foraging opportunities and may also serve as a complementary habitat for species typically associated with estuarine environments. Degradation of reef habitats, for example through eutrophication driven by riverine and groundwater discharges, may therefore reduce the foraging habitat quality available to ariid populations, particularly for brooding males using the reef as refuge. Our findings underscore the importance of integrating estuarine and reef habitats in management frameworks for coastal fishes in Northeastern Brazil and highlight ariid catfishes as valuable bioindicators of reef–estuarine connectivity in this region.

## AUTHOR CONTRIBUTIONS

RGL, BES and APM conceptualized the manuscript and analyses and were primarily responsible for preparing the manuscript. RGL curated the data and conducted the statistical analyses. RGL, BES and APM wrote the original draft, and RGL and BES made the final revisions to the manuscript. APM led the sampling design, sampling efforts and laboratory procedures. ALV led laboratory procedures and contributed to sampling efforts. CACS contributed to the sampling design and created the map. CSP, WRFB, PRS, LKPJ and MVSG processed the fish to obtain dietary and reproductive data. All authors reviewed and proofread the manuscript and provided suggestions and critical feedback.

## Supporting information


**Figure S1.** Total number of individuals sampled by species in Penha Beach, Paraíba, Brazil.
**Figure S2.** Number of individuals sampled per month in all sampled months. Months range from September (Sep) 2023 to August (Aug) 2024. Blue background from March (Mar) to August (Aug) represents wet season.
**Table S1.** Number of individuals for each sex in the different months. NA are individuals that were not possible to identify the sex.
**Figure S3.** Length (mm) weight (g) relationship for males of Bagre filamentosus sampled on dry (pink) and wet (blue) seasons. Both axes are in logarithmic (log10) scale.
**Figure S4.** Alimentary index for the species in dry and wet seasons. Food items were grouped into higher levels.
**Figure S5.** Alimentary index for the species in dry and wet seasons. Food items were grouped into their location. Hard bottom, soft bottom, water (pelagic) and uncertain.
**Figure S6.** Broad overview of the sampled area in Penha beach, Paraíba, northeast of Brazil.

## Data Availability

The data that supports the findings of this study are available in the Supporting Information of this article.
