# Supplementary material for: The habitat use of ariid catfishes in coral reefs in northeastern Brazil
Source: J Fish Biol. 2026 Sep 18;109(5):2776–87. doi: 10.1111/jfb.70654 (PMC13770695; doi:10.1111/jfb.70654)
Supplement: Supplementary file 1 [file JFB-109-2776-s001.docx]

# Supporting information

Figure S 1. Total number of individuals sampled by species in Penha Beach, Paraíba, Brazil.

Figure S 2. Number of individuals sampled per month in all sampled months. Months range from September (Sep) 2023 to August (Aug) 2024. Blue background from March (Mar) to August (Aug) represents wet season.

Table S 1. Number of individuals for each sex in the different months. NA are individuals that were not possible to identify the sex.

| Sample | Year | Month | Season | *Bagre bagre* | | | *Bagre filamentosus* | | | *Cathorops spixii* | | | *Notarius luniscutis* | | | *Notarius parmocassis* | | | *Sciades proops* | | | |  |
| --- | --- | --- | --- | --- | --- | --- | --- | --- | --- | --- | --- | --- | --- | --- | --- | --- | --- | --- | --- | --- | --- | --- | --- |
|  |  |  |  | M | F | NA | M | F | NA | M | F | NA | M | F | NA | M | F | NA | | M | F | NA | |
| 1 | 2023 | September | Dry | 3 | 1 | 0 | 2 | 0 | 0 | 0 | 1 | 0 | 0 | 0 | 0 | 0 | 0 | 0 | | 3 | 1 | 0 | |
| 2 | 2023 | October | Dry | 12 | 1 | 0 | 1 | 0 | 0 | 0 | 0 | 0 | 2 | 2 | 0 | 0 | 0 | 0 | | 3 | 0 | 1 | |
| 3 | 2023 | November | Dry | 0 | 1 | 0 | 11 | 5 | 0 | 0 | 0 | 0 | 0 | 0 | 0 | 0 | 0 | 0 | | 3 | 0 | 0 | |
| 4 | 2023 | December | Dry | 2 | 0 | 0 | 22 | 1 | 0 | 0 | 0 | 0 | 0 | 0 | 0 | 0 | 0 | 0 | | 1 | 2 | 0 | |
| 5 | 2024 | January | Dry | 0 | 0 | 0 | 16 | 12 | 0 | 0 | 0 | 0 | 0 | 0 | 0 | 0 | 0 | 0 | | 0 | 0 | 0 | |
| 6 | 2024 | February | Dry | 0 | 0 | 0 | 6 | 8 | 0 | 0 | 0 | 0 | 0 | 1 | 0 | 0 | 1 | 0 | | 3 | 0 | 0 | |
| 7 | 2024 | March | Wet | 0 | 0 | 0 | 7 | 4 | 2 | 0 | 0 | 0 | 2 | 5 | 1 | 0 | 0 | 0 | | 0 | 1 | 1 | |
| 8 | 2024 | April | Wet | 0 | 0 | 0 | 9 | 0 | 0 | 1 | 2 | 0 | 0 | 3 | 0 | 1 | 0 | 1 | | 1 | 0 | 0 | |
| 9 | 2024 | May | Wet | 0 | 0 | 0 | 10 | 6 | 1 | 4 | 3 | 0 | 2 | 3 | 0 | 0 | 0 | 0 | | 5 | 0 | 0 | |
| 10 | 2024 | June | Wet | 0 | 0 | 0 | 8 | 0 | 0 | 2 | 3 | 1 | 4 | 4 | 0 | 0 | 0 | 0 | | 1 | 0 | 0 | |
| 11 | 2024 | July | Wet | 1 | 0 | 2 | 0 | 0 | 0 | 3 | 0 | 1 | 0 | 0 | 0 | 0 | 0 | 0 | | 0 | 1 | 0 | |
| 12 | 2024 | August | Wet | 0 | 0 | 0 | 11 | 0 | 2 | 0 | 0 | 0 | 0 | 1 | 0 | 0 | 0 | 0 | | 6 | 2 | 0 | |

Figure S 3. Length (mm) weight (g) relationship for males of Bagre filamentosus sampled on dry (pink) and wet (blue) seasons. Both axes are in logarithmic (log10) scale.

Figure S 4. Alimentary index for the species in dry and wet seasons. Food items were grouped into higher levels.

Figure S 5. Alimentary index for the species in dry and wet seasons. Food items were grouped into their location. Hard bottom, soft bottom, water (pelagic) and uncertain.

Figure S 6. Broad overview of the sampled area in Penha beach, Paraíba, northeast of Brazil

**CARTOGRAPHIC BASE**
